# Impurities of crude glycerol and their effect on metabolite production

**DOI:** 10.1007/s13213-013-0767-x

**Published:** 2013-12-13

**Authors:** Dorota Samul, Katarzyna Leja, Włodzimierz Grajek

**Affiliations:** Department of Biotechnology and Food Microbiology, Poznań University of Life Sciences, Wojska Polskiego 48, 60-627 Poznań, Poland

**Keywords:** 1,3-Propanediol, Crude glycerol, Impurities, Pure glycerol

## Abstract

Glycerol is a valuable raw material for the production of industrially useful metabolites. Among many promising applications for the use of glycerol is its bioconversion to high value-added compounds, such as 1,3-propanediol (1,3-PD), succinate, ethanol, propionate, and hydrogen, through microbial fermentation. Another method of waste material utilization is the application of crude glycerol in blends with other wastes (e.g., tomato waste hydrolysate). However, crude glycerol, a by-product of biodiesel production, has many impurities which can limit the yield of metabolites. In this mini-review we summarize the effects of crude glycerol impurities on various microbial fermentations and give an overview of the metabolites that can be synthesized by a number of prokaryotic and eukaryotic microorganisms when cultivated on glycerol.

## Introduction

Glycerol is a valuable raw material for the production of industrially useful metabolites. As such, it has found many applications in the cosmetic, paint, automotive, food, tobacco, pharmaceutical, pulp and paper, leather and textile industries. It is also used as a feedstock for the production of various chemicals (Wang et al. 2001; da Silva et al. 2009). One of many promising applications for the use of glycerol is its bioconversion to high value-added compounds through microbial fermentation. Glycerol is not only cheap and abundant, but its greater degree of reduction than sugars offers the opportunity to obtain reduced chemicals, such as succinate, ethanol, xylitol, propionate, hydrogen, among others, at higher yields than those obtained by using sugars (Dharmadi et al. 2006; da Silva et al. 2009; Leja et al. 2011a). Glycerol is also used in 1,3-propanediol (1,3-PD) synthesis. 1,3-PD is an industrially valuable chemical intermediate with potential applications in the production of polymers (among others, polyesters, polyethers, polyurethanes) (Homann et al. 1990; Zeng and Biebl 2002), cosmetics (Huang et al. 2002), foods, lubricants (Barbirato et al. 1998), medicines (Adachi et al. 1995), and as an intermediate for the synthesis of heterocyclic compounds, such as indoles and quinolones (Tsuji et al. 1987; Zeng et al. 1993; Menzel et al. 1997; Katrlík et al. 2007; Kubiak et al. 2012; Myszka et al. 2012).

Crude glycerol is synthesized as a by-product of biodiesel production. In recent years, biodiesel production has significantly increased in a number of European countries, and according to the U.S. Energy Information Administration issued on July 30, the European Union (EU) produced 111 million gallons in May 2013. This increase in biodiesel production has resulted in a concomitant increase in the amount of crude glycerol produced (Dharmadi et al. 2006; Deckwer 1995). As a consequences some EU biodiesel companies are experiencing severe problems in how to get rid themselves of the excess glycerol as its disposal is quite expensive. The collapse of glycerol prices has also caused a major problem to these companies (Willke and Vorlop 2004; Dharmadi et al. 2006). Thus, solutions to the management of this raw material are badly needed. One possible approach is to use crude glycerol in the production of industrially useful metabolites in fermentation processes. Glycerol is abundant in the natural environment since it is a structural component of many lipids. It is also one of the principal compatible solutes, being widely produced in response to decreased extracellular water activity during osmoregulation in yeasts (Wang et al. 2001; da Silva et al. 2009). Due to its occurrence in nature, many known microorganisms can naturally utilize glycerol as a sole carbon and energy source, including *Klebsiella pneumoniae* (Biebl et al. 1998), *K. oxytoca* (Homann et al. 1990), *K. aerogenes* (Biebl et al. 1998), *Lactobacillus reuterii*, *L. buchnerii*, *L. collinoides* (da Silva et al. 2009), *Enterobacter agglomerans* (Barbirato et al. 1998), *E. aerogenes* (da Silva et al. 2009), *Clostridium butyricum* (Colin et al. 2001), *C. pasteurianum* (Biebl et al. 1992; Dabrock et al. 1992), *C. diolis*, *C. perfringens* (Youngleson et al. 1989; Hao et al. 2008), *Citrobacter freundii* (Boenigk et al. 1993; Daniel et al. 1995; Malinowski 1999), *Pelobacter carbinolicus*, *Rautella planticola*, and *Bacillus welchii* (Saxena et al. 2009; Leja et al. 2011b). Thus, glycerol is a promising and abundant new carbon source for applications in those industrial microbiology where it can substitute for traditional carbohydrates, such as sucrose, glucose and starch, such as in some industrial fermentation processes. However, the use of crude glycerol, without purification, in fermentation is a difficult process. Impurities in this material influence biochemical pathways in bacteria cells and can limit the efficiency of metabolite production. Although utilization of crude glycerol in the fermentation medium without prior purification offers a remarkable advantage against the traditional use of pure glycerol as substrate, there have been only a few published reports describing its use as a sole carbon source (e.g., Adachi et al. 1995; Barbirato et al. 1998; Colin et al. 2001; Dharmadi et al. 2006; Hao et al. 2008; Desbois and Smith 2010; Papanikolaou et al. 2008).

In the study reported here we identified the main impurities present in crude glycerol obtained as a by-product during biodiesel production and compared the yield of metabolites obtained from pure and crude glycerol. Based on these results, we present here a number of possible some solutions on how to limit the negative influence of impurities in crude glycerol.

## Microorganisms able to product industrially useful metabolites from crude glycerol

One of the most promising methods for the valorization of crude glycerol is its use in biotransformation processes for the biosynthesis of high value-added metabolic products. A large group of microorganisms is capable of assimilating glycerol as a carbon source in the synthesis of many useful products, such as 1,3-PD, ethanol, hydrogen, polyhydroxyalkanoates, and organic acids (Amaral et al. 2009; da Silva et al. 2009; André et al. 2010; Chatzifragkou et al. 2011a, b, c). A number of microorganisms can grow on glycerol, including *Actinobacillus succinogenes*, *Aspergillus niger*, *Blakeslea trispora*, *Burkholderia* sp., *Chlorella protothecoides*, *Citrobacter freundii*, *Clostridium buturicum*, *Clostridium pasteurianum*, *Cunninghamella echinulata*, *Cupriavidus necator*, *Enterobacter aerogenes*, *Escherichia coli*, *Gluconobacter* sp., *Klebsiella pneumoniae*, *Kluyvera cryocrescens*, *Lentinula edodes*, *Mortierella ramanniana*, *Mucor* sp., *Pseudomonas oleovorans*, *Rhodotorula glutinis*, *Schizochytrium limacinum*, *Staphylococcus caseolyticus*, *Yarrowia lipolytica*, and *Zobellella denitrificans* (Petitdemange et al. 1995; González-Pajuelo et al. 2004; Hirschmann et al. 2005; Ito et al. 2005; Mu et al. 2006; Rymowicz et al. 2006, 2009; Fakas et al. 2008; Mantzouridou et al. 2008; Volpato et al. 2008; Cavalheiro et al. 2009; Habe et al. 2009; Rywińska et al. 2009; André et al. 2010; Andreeßen et al. 2010; Chatzifragkou et al. 2010; Chee et al. 2010; Ibrahim and Steinbüchel 2010; Liang et al. 2010; Ashby et al. 2011; Choi et al. 2011; O’Grady and Morgan 2011; Saenge et al. 2011; Vlysidis et al. 2011; Bellou et al. 2012; Metsoviti et al. 2012; Venkataramanan et al. 2012; Wilkens et al. 2012). Table 1 presents a number of the microorganisms that are able to convert crude glycerol to commercially useful metabolites as well as transform the main impurities of this raw material..

**Table 1:** Microorganisms able to convert crude glycerol to commercially useful metabolites and the main impurities in crude glycerol having an impact on the conversion of this raw material

Strain	Metabolic product	Glycerol impurities	Reference
*Clostridium butyricum* VPI 1718	1,3-Propanediol (1,3-PD)	Oleic acid	Chatzifragkou et al. 2011a b
*Clostridium butyricum* DSM 15410	1,3-PD	Methanol	Moon et al. 2010
*Klebsiella pneumoniae* DSM 2026	1,3-PD	No data	Mu et al. 2006
*Clostridium pasteurianum* ATCC 6013	1,3-PD	Oleic, linoleic acid	Venkataramanan et al. 2012
*Enterobacter aerogenes* HU-101	Hydrogen	Salts	Ito et al. 2005
*Yarrowia lipolytica* Wratislavia AWG7	Citric acid	Methanol, salts	Rywińska et al. 2009
*Yarrowia lipolytica* N15	Citric acid	Fatty acids	Kamzolova et al. 2011
*Basfia succiniciproducens* DD1	Succinic acid	No data	Scholten et al. 2009
*Gluconobacter* sp. NBRC103465	Glyceric acid	Methanol	Habe et al. 2009
*Yarrowia lipolytica* Wratislavia K1	Erythritol	No data	Rymowicz et al. 2009
*Paracoccus denitrificans*	Polyhydroxyalkanoates	Salts	Mothes et al. 2007
*Cupriavidus necator* DSM 545	Polyhydroxyalkanoates	Sodium salts	Cavalheiro et al. 2009
*Staphylococcus caseolyticus* EX17	Lipase	No data	Volpato et al. 2008
*Cryptococcus curvatus*	Lipid	Methanol	Liang et al. 2010
*Aspergillus niger* NRRL 364	Lipid, oxalic acid	Potassium and sodium salts, non-glycerol organic material, methanol	André et al. 2010
*Lentinula edodes*AMRL 121	Lipid	Potassium and sodium salts, non-glycerol organic material, methanol	André et al. 2010
*Cunninghamella echinulata* ATHUM 4411	Lipid	No data	Fakas et al. 2008
*Mortierella ramanniana*	Lipid	No data	Bellou et al. 2012
*Blakeslea trispora*	β -Carotene	Soap, methanol	Mantzouridou et al. 2008

The transesterification process is carried out during biodiesel (upper phase, nonpolar ester) and crude glycerol (lower phase, polar glycerol) formation. Crude glycerol consists of glycerol, alcohol (typically methanol), inorganic salts (as residues from catalysts), free fatty acid, unreacted mono-, di-and triacylglycerols, methyl esters, and water (Pagliaro and Rossi 2010). The composition of crude glycerol depends mainly on the kind of raw glycerol used for the production of biodiesel, the conditions of the transesterification process, and the conditions of the separate non-polar phase from the polar phase (Hájek and Skopal 2010). The purification of biodiesel is very important, as the quality of the obtained by-product (purity of glycerol) depends on it. The by-products of biodiesel production can contain from 40 to 99 % glycerol depending on the method of purification (Van Gerpen 2005; Leung et al. 2010; Singhabhandhu and Tezuka 2010). The main impurities in crude glycerol are free fatty acid, inorganic salts, and alcohol (Chatzifragkou and Papanikolaou 2012; Venkataramanan et al. 2012). The presence of these impurities in crude glycerol has a very negative effect on the morphology and biochemical processes of bacterial cells and, consequently, lower concentrations of metabolites are obtained than crude glycerol than from pure glycerol (Ito et al. 2005; Wilkens et al. 2012). However, a few examples of the use of crude glycerol with a neutral or positive influence on the synthesis of certain metabolites can be found in the literature (Chatzifragkou et al., 2011a; b; Jun et al. 2010).

Impurities in glycerol interact with each other and can have a synergic effect. Alcohol influences a cell membrane and increases its permeability, but its effect strictly depends on the length of the carbon chain and its concentration. For example, methanol, an aliphatic alcohol with one carbon atom, does not influence the cell membrane (in low concentrations) and does not decrease metabolite production (Venkataramanan et al. 2012). Anther group of glycerol impurities are salts. High concentrations of monovalent salts decrease van der Waals force in a lipid membrane and cause swelling of the cell membrane. This swelling exerts has a negative effect on the energetic barrier in a lipid layer of the membrane and changes the course of biochemical processes in the cells; for example, it influences the transportation of nutrition factors through the cell membrane (Petrache et al. 2006). The free fatty acids (linoleic, stearic, oleic acids) have a major influence on the synthesis of metabolites. Fatty acids are components of the cell membrane and are incorporated into the acyl chains and alk-1-enyl chains of cellular lipids; their presence thus disturbs the correct process of metabolite synthesis (Venkataramanan et al. 2012). Stearic acid is a saturated fatty acid with an 18-carbon chain which is present in the fermentation medium; it aligns with the fatty acid tails of the membrane. Oleic acid consists only of one double bond and it causes a kink in the molecule which hampers the diffusion of nutrient factors and metabolites through the membrane (Furusawa and Koyama 2004). Linoleic acid is a compound with a high degree of unsaturation which has two kinks which cause the inhibition of nutrient factors and thereby limit the synthesis of some products (Desbois and Smith 2010). The unsaturated fatty acids with two or more double bonds which are present in crude glycerol have strong influence on the diffusion of the substrate along the membrane (Venkataramanan et al. 2012).

Crude glycerol is recognized as a renewable waste substrate with numerous applications, and its bioconversion into high value-added metabolic products would provide a valuable option for the efficient management of biodiesel production waste and development of sustainable technologies. The possibility of using crude glycerol as a substrate for the production of important industrial products depends on the degree of its purity. The biotechnological production of 1,3-PD as a valuable by-product of biodiesel production, namely, the production of crude glycerol, is a promising and encouraging alternative to traditional chemical synthesis.

## Comparison of pure and crude glycerol

Although the utilization of raw glycerol in fermentation medium without prior purification is economically much more advantageous than the traditional use of pure glycerol as substrate, only a few reports have appeared in the literature on the use of this substrate as a sole carbon source (Barbirato et al. 1998; González-Pajuelo et al. 2004; Hirschmann et al. 2005; Mu et al. 2006; Rymowicz et al. 2006; Papanikolaou et al. 2008). The biosynthesis of high value-added metabolic products is one of the more promising uses of crude glycerol for biotechnological conversion (Zeng and Sabra 2011; Posada et al. 2012). The final product concentrations and yield of different metabolites from pure and crude glycerol are compared in Table 2.

**Table 2:** Final product concentrations and yield of different metabolites from pure and crude glycerol

Strain	Metabolic product	Final product concentrations from pure glycerol (g/L)	Yield (g/g)	Final product concentrations from crude glycerol (g/L)	Yield (g/g)	Reference
*Clostridium butyricum* AKR102a	1,3PD	93.7	0.52	76.2	0.51	Wilkens et al. 2012
*Citrobacter freundii* FMCC-B 294	1,3-PD	68.1	0.40	66.3	0.38	Metsoviti et al. 2013
*Clostridium butyricum* VPI 1718	1,3-PD	11.3	0.57	11.3	0.57	Chatzifragkou et al. 2011b
*Klebsiella pneumoniae* DSM 4799	1,3-PD	13.8	0.35	17.1	0.42	Jun et al. 2010
*Clostridium butyricum* 2.1.	1,3-PD	31.9	0.46	28.0	0.40	Orczyk and Szymanowska-Powałowska 2012
*Klebsiella pneumoniae* DSM 2026	1,3-PD	61.9	0.41	53	0.39	Mu et al. 2006
*Yarrowia lipolytica* Wratislavia AWG7	Citric acid	19.0	0.69	18.1	0.66	Rywińska et al. 2009
*Yarrowia lipolytica* Wratislavia K1	Citric acid	20.4	0.45	22.1	0.43	Rywińska et al. 2009
*Yarrowia lipolytica* N15	Citric acid	98.0	0.70	71	0.90	Kamzolova et al. 2011
*Aspergillus niger* LFMB 1	Oxalic acid	No data	No data	21.5	0.61	André et al. 2010
*Gluconobacter* sp. NBRC103465	Glyceric acid	54.7	0.33	45.9	0.26	Habe et al. 2009
*Aspergillus niger* NRRL 364	Lipid	No data	No data	3.4	0.21	André et al. 2010
*Gluconobacter* sp. NBRC103465	Dihydroxy-acetone	33.7	0.20	28.2	0.16	Habe et al. 2009
*Staphylococcus caseolyticus* EX17	Lipase	145.8 U/L	No data	127.3 U/L	No data	Volpato et al. 2008
*Cupriavidus necator* DSM 545	Polyhydroxy-alkanoates P(3HB)	51.2	0.36	38.1	0.34	Cavalheiro et al. 2009

In general, there is a larger yield of metabolite product associated with the use of pure glycerol. However, some researchers have reported that the efficiency of the 1,3-PD production process is the same for both types of glycerol (Chatzifragkou et al. 2011b) or even that the efficiency of the production process is higher using crude glycerol (Rywińska et al. 2009; Jun et al. 2010; Jitrwung and Yargeau 2011). One reason for this inconsistency is that the quantity and quality of the composition of the crude glycerol was not analyzed in many studies. In some cases the glycerol was rich in vitamins and other nutrition factors used by bacteria, resulting in higher metabolic activity (Hilaly and Binder 2002). In the study of Jun et al. (2010), the crude glycerol consisted of methanol (0.27 wt%), water (0.05 wt%), non-glycerol organic matter (17.0 wt%), sodium (13.6 mg/kg), potassium (70 mg/kg), and magnesium (1.9 mg/kg). However, these impurities did not decrease the yield of metabolite production—to the contrary, it was higher than when the pure raw material was used. Because many authors do not provide information on the level of impurities in crude glycerol, the quality of glycerol used in the various studies cannot be compared. We can assume that the crude glycerol used in the study of Jun et al. (2010) was good quality glycerol. Additionally, pure glycerol is often purified up to only 80 %; thus, the difference between the raw and pure materials used in some earlier studies was insignificant.

In order to overcome the obstacle of impurities encountered in the composition of the biodiesel-derived crude glycerol utilized, a number of researchers have proposed various pretreatment strategies. In particular, the treatment of diluted crude glycerol with hydrochloric acid precipitates the residual fatty acids, which can be further removed by centrifugation (Moon et al. 2010; Venkataramanan et al. 2012). Moreover, the application of organic solvents (hexane, heptane, octane, petroleum ether, and *n*-hexanol) has been also reported as a means of biodiesel-derived crude glycerol pretreatment for the removal of residual fatty acids (Rehman et al. 2008; Anand and Saxena 2012). Additionally, Anand and Saxena (2012) found that the cultivation of *Citrobacter freundii* on the above-mentioned pretreated glycerol wastes gives results similar to those obtained with pure glycerol in terms of microbial growth and 1,3-PD production, indicating once again the strong negative impact of residual free fatty acids on bacterial growth.

Glycerol as a low-cost by-product of the biodiesel industry can be considered to be a renewable building block for biorefineries. The biosynthesis of high value-added metabolic products from crude glycerol is a current and important issue in the valuation of raw material and use of crude glycerol in many branches of the industry. The development of technology that would allow for the efficient use of by-products of biodiesel production is also an important issue. Effective utilization of crude glycerol is crucial to the commercialization and further development of biodiesel production. The effective utilization of crude glycerol will contribute to the viability of biodiesel.

## Utilization of crude glycerol in blends with other wastes

Industrial fermentations need raw materials that both fulfill the requirements of the organism and are available in a high quantity and quality. Most fermentation media consist of carbon sources, nitrogen sources, minerals and specific nutrients. The carbon to nitrogen ration (C/N) in the growth medium is considered to be the most decisive factor.

There are few published reports on the utilization of crude glycerol in blends with other wastes. Suitable nitrogen sources include, for example, sources of nitrate or ammonium ions, urea, yeast extract, beef extract, proteose peptone, soybean meal, hydrolysates of casein, distiller’s solubles, and the like. Distiller’s solubles can be corn steep liquor, bottom stillage from ethanol distillation, or soybean solubles (Hilaly and Binder 2002).

Tomato waste, comprising tomato skin and seeds, is of particular interest because it is produced in huge amounts in all Mediterranean countries and is a causing severe environmental problems. This pollution could be avoided if there were alternative ways for the valorization of tomato waste. These wastes could then be used as fermentation feedstock to produce microbial lipids (Fakas et al. 2008). Fakas et al. (2008) researched the effect of organic nitrogen on the uptake rate of both glucose and glycerol and observed distinct patterns of lipid accumulation by *Cunninghamella echinulata*. Nevertheless, when glycerol was used as a carbon source instead of glucose, the uptake rate as well as the biomass production and the lipid accumulation processes were unaffected by the removal of organic nitrogen from the tomato waste hydrolysate (TWH). The lipid content in biomass produced on original TWH supplemented with glycerol was lower than that achieved on original TWH supplemented with glucose. Growth of *C. echinulata* was inhibited in TWH media containing glycerol as a carbon source at concentrations of >60 g/L. The glycerol inhibition problem, however, could be resolved by applying fed-batch cultivation systems in which glycerol could be added gradually to the culture. The favorable glycerol conversion yields obtained in this study indicate that glycerol could be an efficient substrate.

The nature of the carbon, a nitrogen source used as raw material for fermentations, depends first on the particular fermentation process and subsequently on the requirements and productivity of the microorganisms. Waste media should be individually evaluated for their capability to support growth microorganisms and metabolite production. Research on simultaneous technology in which wastes from the first step of the process could be used as raw materials in other processes is promising and should be further developed. Supplementation of pure materials by crude raw materials has a tremendous ecological and economic potential for biotechnology processes. This would be of particular interest if TWH and raw glycerol could be used as co-substrates, partly because of the very low (or even negative) economic value of the substrates and partly because this approach could offer the means to recycle, at least partially, the raw glycerol produced during biodiesel manufacturing. Thus, optimization of such processes is necessary. The main problem is that crude materials have unstandardized composition and are of diverse quality depending on the source. Waste media should therefore be individually evaluated for their capability to support growth and metabolite production. Isolation and selection of microorganisms which are able to effectively utilize wastes is a main issue in introducing such processes into industrial applications.

## Conclusion

The production of industrially useful metabolites from crude glycerol is an important issue, as it may be (partially) responsible for the price of biodiesel. A large group of microorganisms are known to produce 1,3-PD, organic acids, and other metabolites. The efficiency of metabolite synthesis depends mainly on the type of glycerol and the microorganisms used. In this report, we have made an attempt to analyze impurities present in crude glycerol, a by-product of biodiesel production, and examine its effect on the efficiency of metabolite production. The solution to this problem is still needed because these impurities limit the up-scaling of fermentation processes and hence it remains impossible to obtain pure products. These purification techniques are very expensive, and alternative methods must therefore be developed. In the Department of Biotechnology and Food Microbiology, Poznań University of Life Sciences, investigations on the production of 1,3-PD from crude glycerol by new isolated *Clostridium butyricum* strain (Orczyk and Szymanowska-Powałowska 2012; Orczyk et al. 2012) are currently being carried out. We are also researching the influence of impurities in crude glycerol on the efficiency of metabolite production. Some data on the utilization of crude glycerol in blends with other waste materials are available. In our opinion, this is a promising technology and should be further tested in our research project in the near future.
